# Modulations of human resting brain connectivity by kisspeptin enhance sexual and emotional functions

**DOI:** 10.1172/jci.insight.121958

**Published:** 2018-10-18

**Authors:** Alexander N. Comninos, Lysia Demetriou, Matthew B. Wall, Amar J. Shah, Sophie A. Clarke, Shakunthala Narayanaswamy, Alexander Nesbitt, Chioma Izzi-Engbeaya, Julia K. Prague, Ali Abbara, Risheka Ratnasabapathy, Lisa Yang, Victoria Salem, Gurjinder M. Nijher, Channa N. Jayasena, Mark Tanner, Paul Bassett, Amrish Mehta, John McGonigle, Eugenii A. Rabiner, Stephen R. Bloom, Waljit S. Dhillo

**Affiliations:** 1 Investigative Medicine, Imperial College London, United Kingdom.; 2 Department of Endocrinology, Imperial College Healthcare NHS Trust, London, United Kingdom.; 3 Imanova Centre for Imaging Sciences, Imperial College London, United Kingdom.; 4 Division of Brain Sciences, Imperial College London, United Kingdom.; 5 Statsconsultancy Ltd, Bucks, United Kingdom.; 6 Department of Neuroradiology, Imperial College Healthcare NHS Trust, London, United Kingdom.; 7 Centre for Neuroimaging Sciences, King’s College London, United Kingdom.

**Keywords:** Endocrinology, Reproductive Biology, Neuroendocrine regulation, Neuroimaging, Sex hormones

## Abstract

**BACKGROUND.** Resting brain connectivity is a crucial component of human behavior demonstrated by disruptions in psychosexual and emotional disorders. Kisspeptin, a recently identified critical reproductive hormone, can alter activity in certain brain structures but its effects on resting brain connectivity and networks in humans remain elusive.

**METHODS.** We determined the effects of kisspeptin on resting brain connectivity (using functional neuroimaging) and behavior (using psychometric analyses) in healthy men, in a randomized double-blinded 2-way placebo-controlled study.

**RESULTS.** Kisspeptin’s modulation of the default mode network (DMN) correlated with increased limbic activity in response to sexual stimuli (globus pallidus **r** = 0.500, **P** = 0.005; cingulate **r** = 0.475, **P** = 0.009). Furthermore, kisspeptin’s DMN modulation was greater in men with less reward drive (**r** = –0.489, **P** = 0.008) and predicted reduced sexual aversion (**r** = –0.499, **P** = 0.006), providing key functional significance. Kisspeptin also enhanced key mood connections including between the amygdala-cingulate, hippocampus-cingulate, and hippocampus–globus pallidus (all **P** < 0.05). Consistent with this, kisspeptin’s enhancement of hippocampus–globus pallidus connectivity predicted increased responses to negative stimuli in limbic structures (including the thalamus and cingulate [all **P** < 0.01]).

**CONCLUSION.** Taken together, our data demonstrate a previously unknown role for kisspeptin in the modulation of functional brain connectivity and networks, integrating these with reproductive hormones and behaviors. Our findings that kisspeptin modulates resting brain connectivity to enhance sexual and emotional processing and decrease sexual aversion, provide foundation for kisspeptin-based therapies for associated disorders of body and mind.

**FUNDING.** NIHR, MRC, and Wellcome Trust.

## Abstract 2

Kisspeptin can modulate resting brain connectivity to enhance sexual and emotional functions in healthy men.

## Introduction

Mammalian reproductive physiology encompasses crucial links between reproductive hormones, brain functions, and behavior, in order to produce and coordinate a complex collection of reproductive functions. The reproductive hormone kisspeptin (encoded by the KISS1 gene), has recently been identified as the master regulator of the reproductive axis, with emerging roles in sexual and emotional behavior (1–6). To this end, kisspeptin and its cognate receptor (encoded by the KISS1R gene) are expressed in key emotional structures of the limbic system in both rodents (7–11) and humans (12–14). Furthermore, we have previously shown that kisspeptin signaling in specific brain structures can modulate downstream reproductive hormone secretion (15), as well as influence human sexual and emotional brain processing during stimulatory tasks (2).

However, the effects of kisspeptin on resting brain networks and connections between these structures, and how this may influence subsequent responses to sexual and emotional stimuli is currently unknown. This is of crucial significance, as resting networks and connections are crucial features of brain function (16), and are sensitive markers of pharmacological effects (17) and clinical conditions (18, 19). Investigating the effects of kisspeptin on resting brain connectivity is therefore important for our understanding of reproductive physiology, as well as the clinical development of kisspeptin-based therapies for common reproductive disorders (20–23). This study was the second part of a large study (part 1 published recently; see ref. 2) investigating the effects of kisspeptin on the human brain, to our knowledge for the first time.

**Figure 1:**
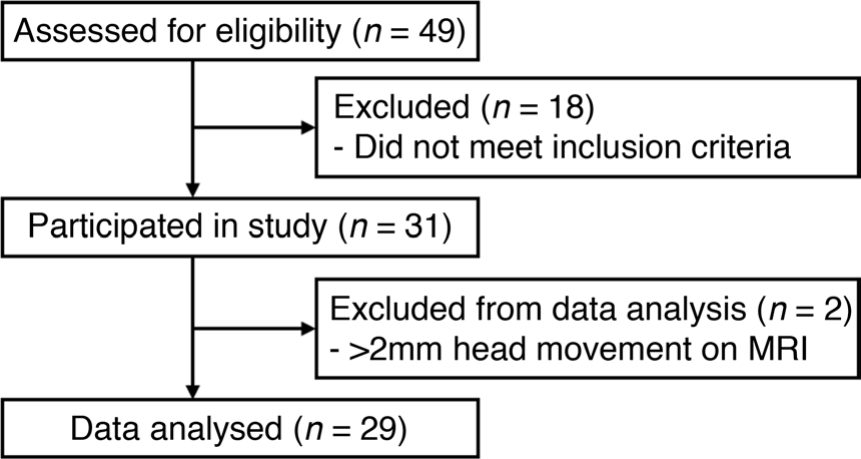
Participant recruitment and flow summary.

We hypothesized that kisspeptin administration modulates resting brain functional connectivity and influences responses to sexual and emotional stimuli. To test our hypotheses, we carried out a randomized, double-blinded, 2-way-crossover, placebo-controlled study in 29 healthy heterosexual young men to investigate the impact of kisspeptin administration on resting-state brain connectivity (Figure 1). In this study, we examined the effects of kisspeptin on resting networks defined using specific a priori limbic and paralimbic seed regions, as well as the 3 major functionally connected large-scale resting networks: the default mode network (DMN), which is associated with social and emotional internal processing, and is disrupted in psychosexual dysfunction (19, 24); the executive control network (ECN), which activates on task performance (24); and the salience network (SN), which selects stimuli that are deserving of our attention (19). Kisspeptin or vehicle was administered and resting brain connectivity was mapped. Kisspeptin’s effects on resting brain connectivity were subsequently correlated to sexual and emotional task response data and psychometric outcomes contained within the same study visits.

## Results

### Kisspeptin did not have a global effect on DMN, ECN, or SN connectivity.

**Figure 2:**
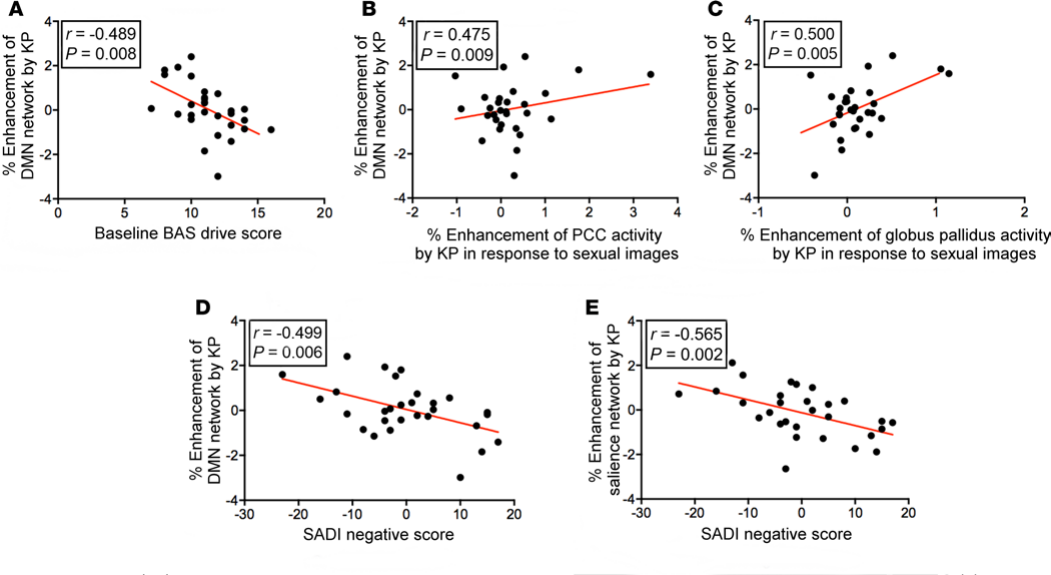
Kisspeptin (KP) modulates resting-state functional connectivity to enhance sexual brain processing and reduce sexual aversion. (**A**) KP administration enhances default mode network (DMN) connectivity more in participants with lower baseline drive reward scores (Behavioral Activation System [BAS] drive). (**B**) KP’s modulation of DMN connectivity correlates with subsequent increases in posterior cingulate cortex (PCC), and (**C**) globus pallidus activity in response to sexual images. (**D** and **E**) KP’s modulation of DMN (**D**) and salience network (**E**) connectivity correlates with reduced sexual aversion (Sexual Arousal and Desire Inventory–Negative). Two-tailed partial correlation testing, adjusted for visit order. *n* = 29 per group.

Group-mean functional connectivity maps for the DMN, ECN, and SN accorded well with previous reports using similar methods (25) (see Supplemental Figures 1 and 2 for group-mean connectivity maps; supplemental material available online with this article; https://doi.org/10.1172/jci.insight.121958DS1). In this study, kisspeptin administration did not affect DMN, ECN, or SN connectivity at a group level. However, individual-level modulations by kisspeptin administration on the DMN and SN were evident, and furthermore correlated with a range of measured outcomes as detailed in turn below (Figure 2 and Supplemental Tables 1–8).

### Kisspeptin’s modulation of the DMN was greater in men with less reward drive.

Kisspeptin administration resulted in greater connectivity of the resting DMN in healthy young men with lower reward drive traits as determined by Behavioral Activation System (BAS) drive score (2-tailed partial correlation adjusted for visit order; *r* = –0.489, *P* = 0.008, Figure 2A and Supplemental Table 1).

### Kisspeptin’s modulation of the DMN correlated with enhancement of limbic activity on viewing sexual images.

Kisspeptin’s modulation of the DMN correlated with greater activity in key limbic structures in response to sexual images (2-tailed partial correlation adjusted for visit order; posterior cingulate *r* = 0.475, *P* = 0.009, Figure 2B; globus pallidus *r* = 0.500, *P* = 0.005, Figure 2C; Supplemental Table 2).

### Kisspeptin’s modulation of the DMN and SN correlated with reduced sexual aversion.

Kisspeptin’s modulation of the DMN correlated with reduced sexual aversion as determined by the Sexual Arousal and Desire Inventory (SADI), providing key functional significance (2-tailed partial correlation adjusted for visit order; *r* = –0.499, *P* = 0.006, Figure 2D and Supplemental Table 1). Furthermore, kisspeptin’s modulation of the SN also correlated with reduced sexual aversion (2-tailed partial correlation adjusted for visit order; *r* = –0.565, *P* = 0.002, Figure 2E and Supplemental Table 5).

### Kisspeptin enhanced global amygdala-cingulate and hippocampus-cingulate connectivity.

**Figure 3:**
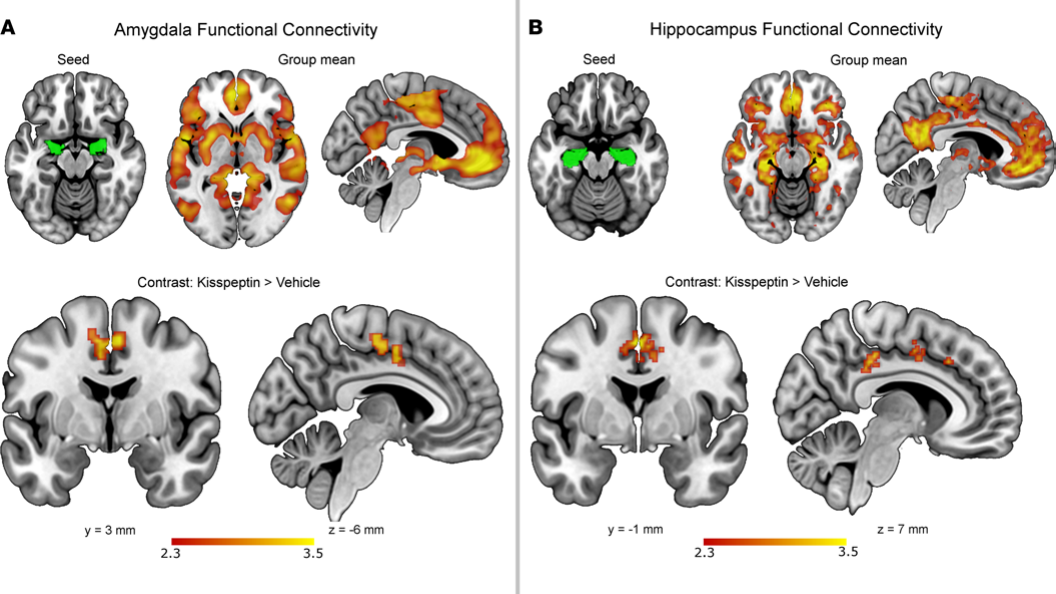
Kisspeptin enhances resting amygdala-cingulate and hippocampus-cingulate global connectivity. (**A**) Employing the amygdala as an anatomically defined seed region (in green) identified increased resting connectivity with the cingulate during kisspeptin administration compared with vehicle. (**B**) Employing the hippocampus as an anatomically defined seed region (in green) identified increased resting connectivity with the cingulate during kisspeptin administration compared with vehicle. Group means for kisspeptin and placebo visits also shown. Whole-brain voxel-wise analyses with cluster correction (*Z* > 2.3, *P* < 0.05). *n* = 29 per group.

Kisspeptin had individual effects on the DMN and SN as detailed above, but did not have a global or regional effect on the DMN, ECN, or SN at a group level. However, we also analyzed the effects of kisspeptin administration on seed-based resting-state networks emanating from a priori anatomically defined limbic structures. We observed that kisspeptin administration enhanced resting amygdala-cingulate (cluster *P* value = 0.035, Figure 3A) and hippocampus-cingulate (cluster *P* values = 2.36 × 10^–8^ and 0.0017, Figure 3B) connectivity.

### Kisspeptin administration enhanced hippocampus–globus pallidus connectivity, which correlated with increased brain responses to negative stimuli in key mood structures.

**Figure 4:**
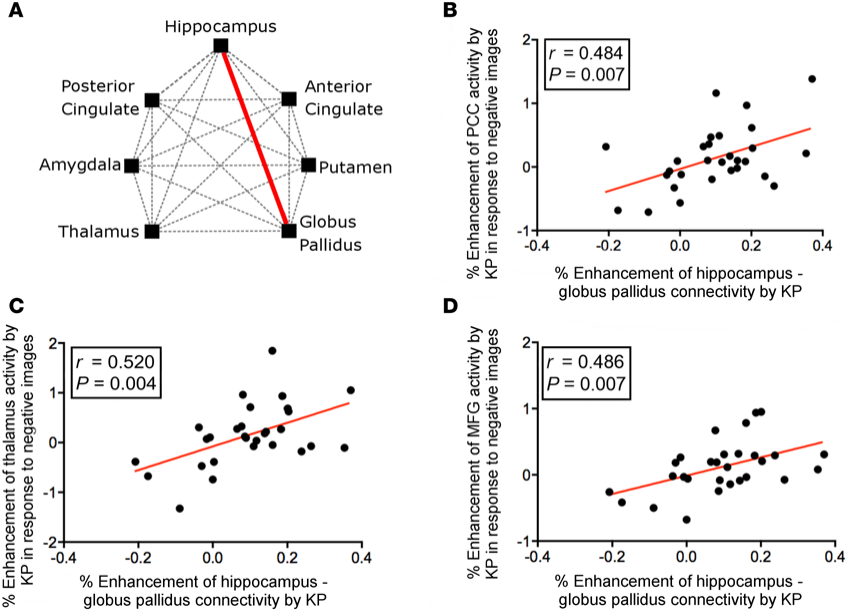
Kisspeptin (KP) enhances hippocampus–globus pallidus ROI connectivity and this predicts brain responses to negative images. (**A**) KP administration enhanced functional connectivity between anatomically defined hippocampus and globus pallidus (*P* = 0.0004). Fisher’s transformation used to convert *r* values into *Z* values followed by 2-tailed paired *t* test with Bonferroni’s correction. *n* = 29 per group. (**B**) KP’s enhancement of hippocampus–globus pallidus connectivity correlated with increased brain activity in the posterior cingulate cortex (PCC), (**C**) thalamus, and (**D**) medial frontal gyrus (MFG). Two-tailed partial correlation testing, adjusted for visit order. *n* = 29 per group.

Finally, we examined the effects of kisspeptin on the interconnections between a priori anatomically defined limbic structures (seed-to-seed analysis). We observed that kisspeptin administration enhanced connectivity between 2 key limbic structures involved in mood regulation, namely the hippocampus and globus pallidus (2-tailed paired *t* test, *t*(28) = –2.662, *P* = 0.0004, Figure 4A). Furthermore, we observed that kisspeptin’s enhancement of their interconnectivity correlated with enhanced brain activity in response to negative images in key mood structures including the posterior cingulate cortex (all 2-tailed partial correlations adjusted for visit order; *r* = 0.484, *P* = 0.007, Figure 4B and Supplemental Table 8), thalamus (*r* = 0.520, *P* = 0.004, Figure 4C and Supplemental Table 8), medial frontal gyrus (MFG) (*r* = 0.486, *P* = 0.007, Figure 4D and Supplemental Table 8), putamen (*r* = 0.485, *P* = 0.007, Supplemental Table 8), nucleus accumbens (*r* = 0.475, *P* = 0.009, Supplemental Table 8), and the caudate (*r* = 0.508, *P* = 0.005, Supplemental Table 8).

## Discussion

In this study, we provide the first evidence to our knowledge that the reproductive hormone kisspeptin modulates resting brain connectivity, and this is associated with enhanced subsequent responses to sexual and negative stimuli, as well as reduced sexual aversion.

The physiological function of reproductive hormones is not only to provide mature gametes, but also to promote positive behaviors that ensure appropriate use of these gametes. These behaviors involve a complex interplay between factors such as sexual arousal, sexual aversion, and mood. We have previously demonstrated that kisspeptin administration to healthy men enhances activity in specific brain structures in response to sexual and bonding stimuli and that this activity correlates with associated mood and behavior (2). However, it is their interconnections and networks in the brain that provide a crucial baseline level of brain function (16–18), and it is well established that this can influence subsequent responses to stimuli, as they share common pathways (26, 27). We believe this is the first study to examine the effects of kisspeptin on these functional connections and networks in humans and correlate them with psychometric outcomes to provide functional relevance.

The DMN is the principal resting-state network, and is predominantly active during internalized thought, such as mental simulation and the processing of emotional stimuli (19). In this study, we demonstrate that individual DMN responses to kisspeptin correlate with a range of subsequent brain responses and behavioral measures. Firstly, kisspeptin’s modulation of the resting DMN was greater in participants less driven to reward. DMN connectivity is known to be lower in individuals with lesser reward traits (28). Therefore, our data suggest that kisspeptin may heighten DMN activity in less reward–driven individuals to potentially enhance internalized thought, emotions, and other DMN functions that may relate to reproductive behaviors. Secondly, we observed that kisspeptin’s modulation of resting brain DMN connectivity at an individual level correlated positively with subsequent increased responses to sexual stimuli in key limbic structures involved in emotional and sexual processing (including the posterior cingulate cortex and globus pallidus). This is particularly relevant, as it has previously been shown that DMN and SN connectivity is disrupted in psychosexual disorders (19), and so our data suggest that kisspeptin may be able to improve DMN connectivity in relation to limbic reward and sexual processing. To this end, and providing key functional relevance, we demonstrate that kisspeptin’s modulation of both the DMN and SN at an individual level correlates with reduced sexual aversion. Collectively, these data suggest that kisspeptin can modulate major functionally connected resting-state networks to enhance responses to sexual stimuli and reduce sexual aversion. Furthermore, these data, coupled with evidence of impaired DMN and SN connectivity in psychosexual disorders (19), raise the intriguing possibility that with further study kisspeptin-based therapies may have clinical applications in patients with psychosexual disorders.

In addition to the DMN and SN, we also examined the effects of kisspeptin on resting global connectivity from key limbic structures. Here we observed that kisspeptin enhanced connectivity between the amygdala-cingulate and hippocampus-cingulate. The amygdala-cingulate network has crucial roles in emotional regulation (29), including depression (30) and bonding (31), while the hippocampus has similar roles and also acts in concert with the amygdala in emotional memory (32). Therefore, our current data demonstrating kisspeptin-mediated enhancement of these established emotional brain connections suggest further roles for kisspeptin in associated emotions.

Finally, we sought to examine the resting connections between a priori anatomically defined limbic structures. We observed that kisspeptin markedly enhanced connectivity between the hippocampus and globus pallidus, two structures that are frequently reported to be anatomically and functionally abnormal in clinical depression (33–35). This is particularly intriguing, as we observed that kisspeptin’s enhancement of hippocampus–globus pallidus connectivity correlated with increased brain responses in key negative mood structures including the posterior cingulate cortex (PCC), thalamus, MFG, and nucleus accumbens. Taken in combination with established reduction of negative mood by kisspeptin administration in animals (36) and humans (2), as well as kisspeptin’s enhancement of MFG activity in response to negative stimuli (2), these data suggest that kisspeptin can modulate both the resting and active brain in relation to negative mood, a finding with exciting clinical implications.

It is interesting to consider the mechanisms by which kisspeptin can exert these effects. We have previously demonstrated that peripherally administered kisspeptin-54 can cross the blood-brain-barrier (BBB), providing access to central kisspeptin receptors and other associated neuroendocrine pathways (2). Previous rodent work suggests that central kisspeptin can also interact with several behavioral neuroendocrine pathways including the serotonergic, adrenergic, vasopressinergic, and dopaminergic systems (1, 6) as well as neuropeptide FF receptors (37, 38).

Another possible mechanistic pathway to consider is that the observed effects on behavioral brain processing in the current study may be in some part due to kisspeptin activation of gonadotropin-releasing hormone (GnRH) neurons (39), which are also implicated in a variety of social behaviors (40, 41). Indeed, kisspeptin does not even need to cross the BBB to access GnRH neurons, as these neurons extend dendrites outside the BBB in specific circumventricular organs such as the organum vasculosum laminae terminalis (OVLT) (42). However, data from our previous study (2) and data from other groups (43) demonstrate that the isoform kisspeptin-54 (used in the current study) is also able to cross the BBB.

Recently, Helier, Brock, and colleagues addressed kisspeptin’s downstream mechanistic pathways and their dependence on GnRH signaling in certain rodent reproductive behaviors (3). This study demonstrates that while kisspeptin’s essential role in olfactory male-directed mate preference is dependent on GnRH, by contrast kisspeptin’s role in lumbar lordosis (a key precopulatory female reproductive behavior) is independent of GnRH. This was evidenced by the failure of GnRH administration to restore lumbar lordosis behavior in kisspeptin-knockout rodents, as well as the finding of preserved lordosis behavior in adult rodents unable to secrete GnRH (3). Interestingly, this pivotal study revealed that kisspeptin’s control of both of these behaviors (mate preference and lordosis) is mediated by hypothalamic neurons expressing neuronal nitric oxide synthase (nNOS) acting downstream of kisspeptin neurons. These hypothalamic nNOS neurons are also pivotal in the control of reproductive hormones (44, 45). Collectively, these data suggest an essential role for both kisspeptin and nitric oxide in these rodent reproductive behaviors, with GnRH also essential in olfactory-driven mate preference but not lumbar lordosis. Although our study examined a different species and a different set of behavioral processes, it is entirely feasible that nitric oxide and GnRH may play a role in at least some of the observed effects. Studies employing GnRH agonists/antagonists and nitric oxide donors could prove fruitful to elucidate this further.

Taken together, it is clear that kisspeptin’s ability to influence a broad range of resting and active sexual and emotional processes relies on its ability to interact with a wide range of different neuroendocrine pathways. This will no doubt be the subject of intense future investigation, particularly in the hope of manipulating these pathways for patients with associated pathologies. Indeed, this study provides the foundation that kisspeptin-based therapies may have clinical applications in psychosexual and depressive disorders, both of which may be intriguing avenues for further study. For example, psychosexual dysfunction affects 1 in 3 people with profound consequences for individuals and their partners, impairing quality of life and chances of successful conception, while currently available treatments are often limited by side-effects (including potential risk of cardiovascular events and cancer; see refs. 46–48) and frequently do not fully restore psychosexual function, particularly when eugonadal (49). Therefore, this study addresses the unmet need to better understand human sexual and emotional processing to potentially inform novel treatments for associated pathologies.

In this study, we advance on previous work exploring the effects of kisspeptin on specific brain structures (2), by investigating kisspeptin’s modulations of important human resting brain connections and networks and correlating these to functional outcomes. Our current data provide evidence that kisspeptin modulates resting brain connectivity, and this enhances responses to sexual and negative stimuli, as well as reducing sexual aversion. We therefore demonstrate a previously undescribed role for kisspeptin in the integration of resting brain connectivity, sexual and emotional processing, sexual aversion, and reproductive hormones in humans. This study has important implications in humans for our understanding of how reproductive hormones not only ensure gametogenesis, but also orchestrate appropriate behaviors that favor sexual activity and ultimately reproduction to ensure survival of the species at a population level. Furthermore, our data provide scientific foundation for the investigation of kisspeptin-based therapies for related psychosexual and emotional disorders, as well as informing ongoing work in the field of in vitro fertilization (20) and other common reproductive disorders such as hyperprolactinemia (22).

## Methods

### Participants.

Thirty-one healthy young men were recruited following a medical screening appointment. Participants were free of present or past physical or psychiatric illness and were naive to psychoactive substances for a minimum of 6 months prior to screening. Participants were excluded if there was a history of sexual aggression/abuse/phobia or psychotherapy/counseling.

Two participants were excluded due to excessive head movement during fMRI scanning (a priori, >2 mm), resulting in a final study group of 29 healthy young men (age 25.0 ± 0.9 years, BMI 23.6 ± 0.4 kg/m^2^, 25 right-handed, 4 left-handed). They had normal physical examination and blood tests for full blood cell count, renal function, liver function, and basal reproductive hormone levels (luteinizing hormone [LH] 3.6 ± 0.3 IU/l, follicle-stimulating hormone [FSH] 2.8 ± 0.2 IU/l, testosterone 21.3 ± 1.2 nmol/l). Electrocardiograms were within normal limits.

### Sample size.

The sample size was selected to allow sufficient power to detect a difference in fMRI brain activity during a hormonal intervention compared with vehicle. This was based on empirically derived estimates of sample sizes in fMRI studies (50) and our previous hormone-fMRI work (51).

### Study design.

We performed a randomized, double-blinded, 2-way-crossover, placebo-controlled study. This study was the second part of a study examining the effects of kisspeptin administration on brain activity. The first part examined the effects of kisspeptin on task-based brain activity (2). In this second part, we examined the effects of kisspeptin on resting-state brain activity. We therefore detail the resting-state components pertinent to the current resting-state study and summarize the shared methods with the first part.

Participants attended 2 study visits each, one for administration of kisspeptin and one for administration of vehicle. In this way, participants acted as their own controls, thereby minimizing interparticipant variations in normal healthy physiology.

Participants were asked to abstain from alcohol, caffeine, and tobacco from midnight and had a normal breakfast on study days. Studies commenced in the morning to ensure peak basal reproductive hormone levels.

On arrival, participants were asked to relax and complete a set of psychometric questionnaires (as described in ref. 2). These included the Behavioral Inhibition System (BIS) scale to assess sensitivity to anticipation of punishment (52), the BAS scale to assess sensitivity to reward, desired goals, and fun (52), the SADI to assess multidimensional sexual arousal and desire (53), and the Positive and Negative Affect Schedule (PANAS) to assess a variety of positive and negative emotions and feelings (54).

An intravenous cannula was sited in each antecubital fossa, one for administration of kisspeptin/vehicle and one for blood collection at 15-minute intervals (assayed as previously described in ref. 2). At t = 0 minutes, a 75-minute infusion of either kisspeptin or vehicle was commenced. Participants and fMRI data analysts (authors LD and MBW) were blinded as to the infusion identity, which was randomized by an independent investigator (using www.randomizer.org).

An intravenous dose of 1 nmol/kg/hr of kisspeptin-54 was selected to provide steady-state levels of circulating kisspeptin from 30 to 75 minutes (during fMRI scanning and questionnaires) while avoiding any testosterone increase in this timeframe as previously demonstrated (55, 56). Kisspeptin-54 (Bachem) was made up in gelofusine (B. Braun) and infused as previously described (56). Vehicle (gelofusine) was administered at an equivalent rate to the kisspeptin infusion.

Between 30 and 75 minutes of the kisspeptin or vehicle infusion, participants underwent fMRI scanning consisting of a resting-state scan and task-response scans (see below), as well as a further set of psychometric questionnaires (2).

### MRI protocol.

The complete MRI session consisted of a localizer, a high-resolution T1-weighted anatomical image, a B0 field-map image, a resting-state fMRI scan, an emotional images task, an emotional faces task, and an fMRI battery task as previously described (2). For the purposes of the functional tasks and open-eyes resting-state scan a mirror was mounted on the head coil to view a screen mounted in the rear of the scanner bore, where visual stimuli were back-projected. The resting-state scan lasted 8 minutes, during which participants were instructed to keep their eyes open and focus on a gray cross on the screen.

### MRI acquisition.

All scanning was performed on a 3T Siemens Trio scanner with a 32-channel phased-array head coil. Anatomical images were acquired at the beginning of each scan using a T1-weighted MPRAGE pulse sequence (1 mm isotropic voxels, TR = 2,300 ms, TE = 2.98 ms, flip angle = 9°). Functional images for the resting-state scan were acquired using a 3D echo planar imaging (EPI) sequence with the following parameters: TR = 2,000 ms, TE1 = 13 ms, TE2 = 31 ms, flip angle = 80°, 36 axial slices, voxel size = 3 mm isotropic and 240 volumes.

### fMRI data analysis.

Functional and structural data were processed using a custom pipeline constructed using 3 complementary image software packages: FMRIB Software Library (FSL), Analysis of Functional Neuroimages (AFNI), and Advanced Normalization Tools (ANTS). The pre-processing included de-spiking (AFNI), slice timing correction (AFNI), motion correction (framewise displacement; AFNI), brain extraction (brain extraction tool in FSL), nonlinear spatial normalization (ANTS), spatial smoothing (6-mm FWHM; AFNI), band-pass filtering (0.01 to 0.08 Hz; AFNI), linear and quadratic detrending (AFNI), and regression of nuisance signals (6 motion parameters, ventricles, draining veins, and local white matter; 25-mm radius; FSL). All statistical analyses of the brain images were then performed using FSL’s FEAT module (see below).

### Seed-based resting-state networks.

Based on a priori hypotheses, the synchrony between anatomically defined seed regions and the whole brain was examined. These were the amygdala, hippocampus, anterior and posterior cingulate, thalamus, globus pallidus, and putamen, based on the expression pattern of KISS1/KISS1R in the limbic and paralimbic system in humans (12, 13) and established structures involved in sexual and emotional processing (18, 19, 24, 28–35, 57). These seed regions were defined anatomically using the Harvard-Oxford subcortical atlas included with FSL. The posterior cingulate, dorso-lateral prefrontal cortex (DLPFC), and anterior insula masks were derived from meta-analytic data for the default mode, executive control, and salience network terms, respectively, on Neurosynth (http://www.neurosynth.org/). The regions were manually isolated from the Neurosynth data, and binary masks were created that retained the cluster morphology of the original images for the specific region required. Time-series were extracted from these regions for use in the functional connectivity analyses. First-level analyses used these time series as the regressor of interest. Subsequently, higher-level analyses were performed to compare kisspeptin with vehicle conditions using FSL’s FEAT to perform a mixed-effects cluster-corrected (*Z* > 2.3, *P* < 0.05) analysis (including a regressor to model visit order). Network definitions were as follows (as previously published in ref. 25). Positive connectivity with the posterior cingulate region defined the DMN (58, 59). Positive connectivity with the DLPFC defined the ECN (60–62). Positive connectivity with the anterior insula defined the SN (61, 63).

### Seed-to-seed analysis.

Region of interest (ROI) analysis was performed on the a priori defined set of limbic and paralimbic regions as above. Correlations against these ROIs and mood structures were run on the ROI time series for each participant and visit.

### Statistics.

Statistical analyses were performed in collaboration with a statistician (PB). For all group-level seed-based voxelwise analyses, a cluster-corrected threshold of *Z* > 2.3, *P* < 0.05 was used. Synchrony between ROIs in the seed-to-seed analysis was assessed using 2-tailed Pearson correlations, and Fisher’s transformation was then used to convert the *r* values to *Z* scores. Kisspeptin and vehicle conditions were subsequently compared using 2-tailed paired *t* tests with Bonferroni’s correction. Psychometric data were normally distributed by Kolmogorov testing. Correlations between brain activity (global signal from the entire networks derived from the seed-based analyses, and synchrony measures from the seed-to-seed analysis) and psychometric outcomes were assessed using 2-tailed partial correlation with associations adjusted for visit order. An α threshold of *P* < 0.05 identified statistical significance, except for the latter correlation analyses, where the threshold was reduced to *P* < 0.01 (to adjust for the number of analyses as previously published in ref. 2).

### Study approval.

The study was approved by the regional ethics committee (National Research Ethics Service London, United Kingdom, REC ref: 04/Q0406/151). The study was performed in accordance with the Declaration of Helsinki, and participants gave written informed consent prior to study inclusion.

## Author contributions

ANC, LD, MBW, CNJ, EAR, SRB, and WSD conceived the study. ANC, LD, MBW, AJS, SAC, SN, AN, CIE, JKP, AA, RR, LY, VS, GMN, and MT collected the data. ANC, LD, MBW, AJS, PB, AM, and JM analyzed the data. ANC, LD, MBW, and WSD wrote the manuscript.

## Supplementary Material

Supplemental data

ICMJE disclosure forms

## Version 1 10/18/2018

Electronic publication
