# Supplementary material for: Modulations of human resting brain connectivity by kisspeptin enhance sexual and emotional functions
Source: JCI Insight. 2018 Oct 18;3(20):e121958. doi: 10.1172/jci.insight.121958 (PMC6237465; doi:10.1172/jci.insight.121958)
Supplement: Supplemental data [file jciinsight-3-121958-s034.pdf]

Supplemental Figure 1. Group mean spatial maps. Group mean for all participants (*n*=29) for both visits (kisspeptin and vehicle) for the following seed-based resting state networks: (A) Default mode (DMN), (B) Executive control (ECN), (C) Salience (SAL), (D) Amygdala, (E) Hippocampus, (F) Anterior cingulate, (G) Putamen, (H) Globus pallidus, (I) Thalamus. All analyses performed using cluster-based thresholding (Z = 2.3, *P* < 0.05) for the purposes of inference. Spatial maps thresholded at Z = 4 here for the purposes of clarity of display.

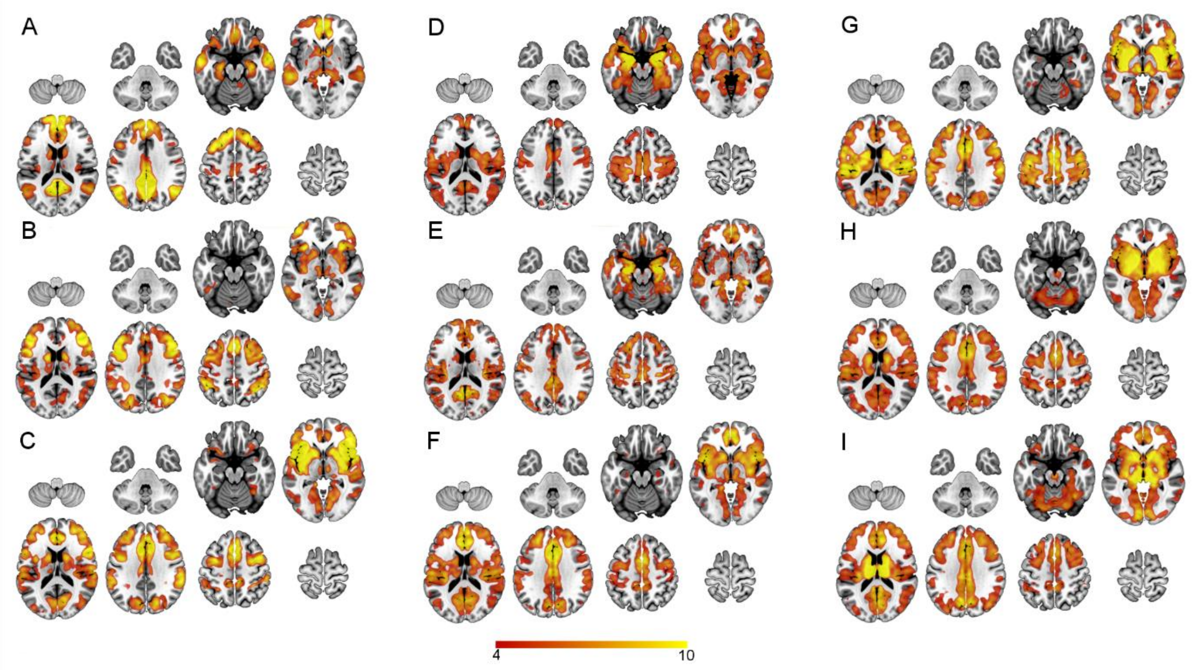

Supplemental Figure 2. Overlap between seed-based and Neurosynth networks. Group mean spatial maps for all participants (*n*=29) for both visits (kisspeptin and vehicle) using seed-based analysis (in blue) overlaid on the respective networks from Neurosyth meta-analysis tool (http://www.neurosynth.org/; in red-yellow) for (A) Default mode (DMN), (B) Executive control (ECN) and (C) Salience (SAL). Spatial maps are thresholded for clarity of display (Z = 2).

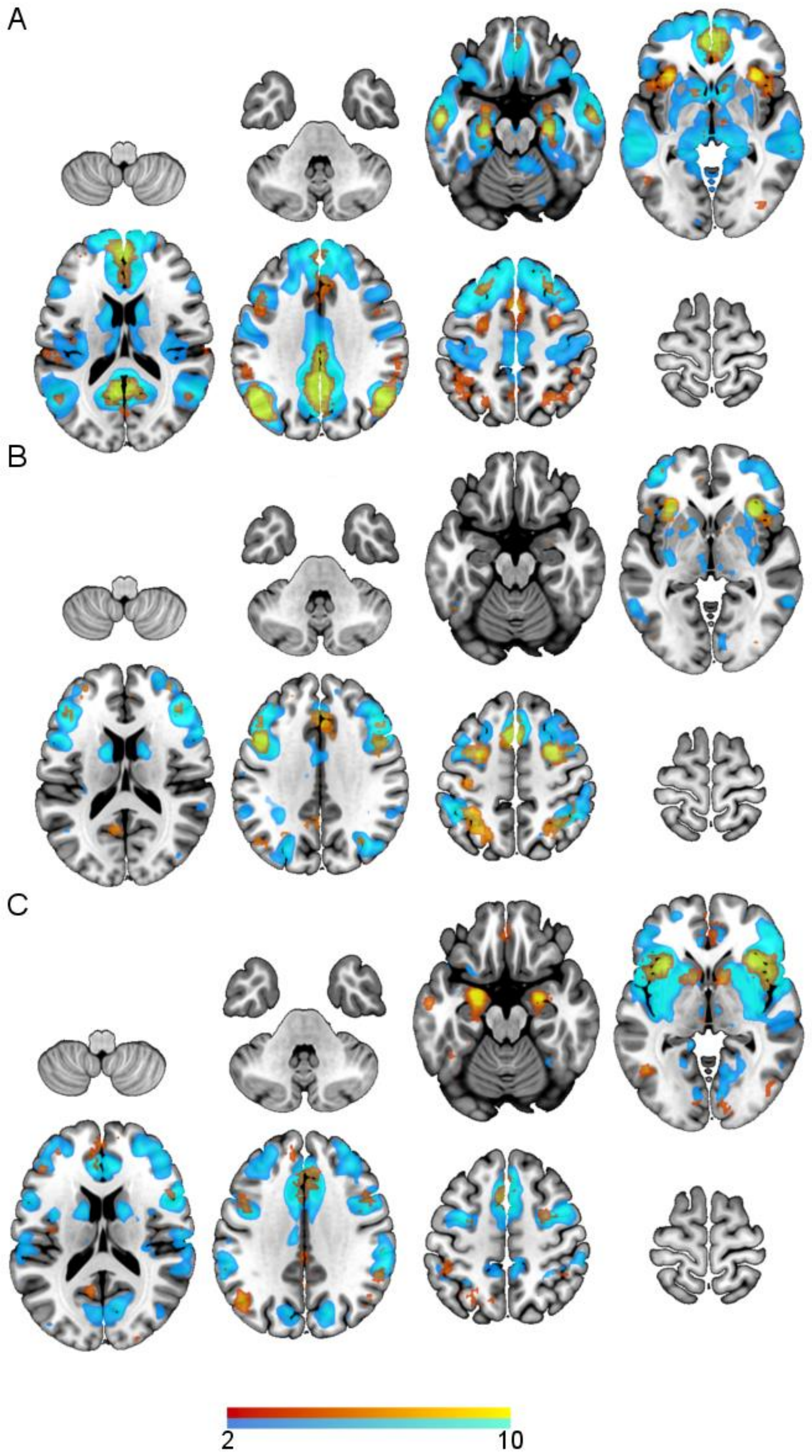

Supplemental Table 1. Correlation analyses between kisspeptin's modulation of the Default Mode Network (DMN) connectivity with psychometric outcomes. Two-tailed partial correlations with associations adjusted for visit order as previously described (1). ** *P* < 0.01. *n*=29.

	Correlation Coefficient (r), *P* Value
BAS Drive	-0.489, 0.008**
BAS Fun	-0.176, 0.368
BAS Reward	-0.079, 0.691
BIS	0.109, 0.582
SADI Evaluative	0.002, 0.993
SADI Negative	-0.499, 0.006**
SADI Physiological	-0.112, 0.570
SADI Motivational	0.072, 0.716
Positive Affect Change	-0.072, 0.714
Negative Affect Change	0.056, 0.777

Supplemental Table 2. Correlation analyses between kisspeptin's modulation of the Default Mode Network (DMN) connectivity with task brain activity in *a priori* limbic and paralimbic brain structures. Two-tailed partial correlations with associations adjusted for visit order as previously described (1). ** $P < 0.01$. n=29.

		Correlation Coefficient (r), *P* Value
Amygdala	Sexual	0.273, 0.151
	Bonding	-0.181, 0.347
	Negative	0.253, 0.185
	Neutral	0.106, 0.585
Hippocampus	Sexual	0.275, 0.148
	Bonding	0.041, 0.832
	Negative	0.279, 0.142
	Neutral	0.346, 0.065
Thalamus	Sexual	0.313, 0.097
	Bonding	-0.115, 0.552
	Negative	0.232, 0.224
	Neutral	0.219, 0.253
Putamen	Sexual	0.406, 0.028
	Bonding	-0.063, 0.747
	Negative	0.230, 0.229
	Neutral	0.256, 0.179
Globus Pallidus	Sexual	0.500, 0.005**
	Bonding	-0.094, 0.628
	Negative	0.185, 0.335
	Neutral	0.145, 0.451
Anterior Cingulate	Sexual	0.338, 0.072
	Bonding	-0.206, 0.283
	Negative	0.139, 0.473
	Neutral	0.113, 0.560
Posterior Cingulate	Sexual	0.475, 0.009**
	Bonding	-0.239, 0.211
	Negative	0.271, 0.154
	Neutral	0.139, 0.473
Medial Frontal Gyrus	Sexual	0.298, 0.115
	Bonding	-0.094, 0.628
	Negative	0.185, 0.335
	Neutral	0.062, 0.747
Nucleus Accumbens	Sexual	0.130, 0.500
	Bonding	-0.220, 0.251
	Negative	-0.039, 0.841
	Neutral	-0.008, 0.967
Caudate	Sexual	0.149, 0.441
	Bonding	-0.354, 0.059
	Negative	-0.051, 0.794
	Neutral	-0.057, 0.771

Supplemental Table 3. Correlation analyses between kisspeptin's modulation of the Executive Control Network (ECN) connectivity with psychometric outcomes. Two-tailed partial correlations with associations adjusted for visit order as previously described (1). ** $P < 0.01$. n=29.

	Correlation Coefficient (r), *P* Value
BAS Drive	0.263, 0.177
BAS Fun	0.032, 0.870
BAS Reward	0.277, 0.153
BIS	-0.005, 0.980
SADI Evaluative	0.148, 0.453
SADI Negative	-0.026, 0.897
SADI Physiological	0.047, 0.812
SADI Motivational	0.015, 0.938
Positive Affect Change	-0.085, 0.668
Negative Affect Change	0.031, 0.877

Supplemental Table 4. Correlation analyses between kisspeptin's modulation of the Executive Control Network (ECN) connectivity with task brain activity in *a priori* limbic and paralimbic brain structures. Two-tailed partial correlations with associations adjusted for visit order as previously described (1). ** $P < 0.01$. n=29.

		Correlation Coefficient (r), *P* Value
Amygdala	Sexual	-0.224, 0.242
	Bonding	-0.310, 0.102
	Negative	-0.198, 0.302
	Neutral	-0.098, 0.614
Hippocampus	Sexual	-0.304, 0.109
	Bonding	-0.398, 0.032
	Negative	-0.294, 0.121
	Neutral	-0.132, 0.495
Thalamus	Sexual	-0.286, 0.133
	Bonding	-0.325, 0.085
	Negative	-0.339, 0.072
	Neutral	-0.204, 0.289
Putamen	Sexual	-0.229, 0.231
	Bonding	-0.335, 0.076
	Negative	-0.306, 0.106
	Neutral	-0.355, 0.058
Globus Pallidus	Sexual	-0.271, 0.156
	Bonding	-0.115, 0.552
	Negative	-0.186, 0.334
	Neutral	-0.119, 0.538
Anterior Cingulate	Sexual	-0.155, 0.422
	Bonding	-0.067, 0.730
	Negative	-0.166, 0.390
	Neutral	0.008, 0.966
Posterior Cingulate	Sexual	-0.230, 0.231
	Bonding	-0.170, 0.378
	Negative	-0.001, 0.996
	Neutral	-0.166, 0.390
Medial Frontal Gyrus	Sexual	-0.233, 0.224
	Bonding	-0.284, 0.136
	Negative	-0.346, 0.066
	Neutral	-0.196, 0.307
Nucleus Accumbens	Sexual	-0.104, 0.592
	Bonding	-0.165, 0.393
	Negative	-0.056, 0.773
	Neutral	0.093, 0.631
Caudate	Sexual	-0.295, 0.121
	Bonding	-0.334, 0.077
	Negative	-0.318, 0.093
	Neutral	-0.255, 0.182

Supplemental Table 5. Correlation analyses between kisspeptin's modulation of the Salience Network (SN) connectivity with psychometric outcomes. Two-tailed partial correlations with associations adjusted for visit order as previously described (1). ** $P < 0.01$. n=29.

	Correlation Coefficient (r), *P* Value
BAS Drive	-0.264, 0.173
BAS Fun	0.020, 0.918
BAS Reward	0.040, 0.841
BIS	-0.143, 0.467
SADI Evaluative	0.191, 0.328
SADI Negative	-0.565, 0.002**
SADI Physiological	0.073, 0.712
SADI Motivational	0.267, 0.168
Positive Affect Change	0.109, 0.582
Negative Affect Change	0.119, 0.545

Supplemental Table 6. Correlation analyses between kisspeptin's modulation of the Salience Network (SN) connectivity with task brain activity in *a priori* limbic and paralimbic brain structures. Two-tailed partial correlations with associations adjusted for visit order as previously described (1). ** $P < 0.01$. n=29.

		Correlation Coefficient (r), *P* Value
Amygdala	Sexual	0.087, 0.654
	Bonding	-0.063, 0.747
	Negative	0.107, 0.579
	Neutral	-0.018, 0.926
Hippocampus	Sexual	0.148, 0.442
	Bonding	0.124, 0.522
	Negative	0.196, 0.307
	Neutral	0.187, 0.332
Thalamus	Sexual	0.140, 0.469
	Bonding	-0.137, 0.479
	Negative	0.072, 0.711
	Neutral	0.008, 0.967
Putamen	Sexual	0.221, 0.248
	Bonding	-0.023, 0.904
	Negative	0.114, 0.555
	Neutral	0.138, 0.474
Globus Pallidus	Sexual	0.291, 0.124
	Bonding	-0.056, 0.774
	Negative	0.030, 0.878
	Neutral	0.003, 0.987
Anterior Cingulate	Sexual	0.164, 0.394
	Bonding	-0.145, 0.453
	Negative	0.158, 0.413
	Neutral	0.024, 0.901
Posterior Cingulate	Sexual	0.301, 0.112
	Bonding	-0.063, 0.744
	Negative	0.237, 0.215
	Neutral	0.158, 0.413
Medial Frontal Gyrus	Sexual	0.024, 0.900
	Bonding	-0.207, 0.281
	Negative	-0.059, 0.761
	Neutral	-0.178, 0.354
Nucleus Accumbens	Sexual	-0.107, 0.580
	Bonding	-0.144, 0.456
	Negative	-0.139, 0.470
	Neutral	0.020, 0.918
Caudate	Sexual	-0.079, 0.685
	Bonding	-0.268, 0.159
	Negative	-0.175, 0.362
	Neutral	-0.106, 0.583

Supplemental Table 7. Correlation analyses between kisspeptin's modulation of hippocampus-globus pallidus connectivity with psychometric outcomes. Two-tailed partial correlations with associations adjusted for visit order as previously described (1). ** $P < 0.01$. n=29.

	Correlation Coefficient (r), *P* Value
BAS Drive	0.245, 0.207
BAS Fun	-0.027, 0.892
BAS Reward	0.207, 0.289
BIS	0.307, 0.111
SADI Evaluative	-0.226, 0.246
SADI Negative	-0.313, 0.103
SADI Physiological	-0.286, 0.138
SADI Motivational	-0.055, 0.782
Positive Affect Change	-0.019, 0.922
Negative Affect Change	0.090, 0.647

Supplemental Table 8. Correlation analyses between kisspeptin's modulation of hippocampus-globus pallidus connectivity with task brain activity in *a priori* limbic and paralimbic brain structures. Two-tailed partial correlations with associations adjusted for visit order as previously described (1). ** $P < 0.01$. n=29.

		Correlation Coefficient (r), *P* Value
Amygdala	Sexual	0.134, 0.487
	Bonding	0.164, 0.396
	Negative	0.353, 0.059
	Neutral	0.034, 0.862
Hippocampus	Sexual	0.131, 0.497
	Bonding	0.175, 0.362
	Negative	0.423, 0.021
	Neutral	0.158, 0.411
Thalamus	Sexual	0.159, 0.409
	Bonding	0.144, 0.456
	Negative	0.520, 0.004**
	Neutral	0.290, 0.125
Putamen	Sexual	0.293, 0.122
	Bonding	0.269, 0.158
	Negative	0.485, 0.007**
	Neutral	0.360, 0.054
Globus Pallidus	Sexual	0.209, 0.276
	Bonding	0.106, 0.584
	Negative	0.462, 0.011
	Neutral	0.227, 0.236
Anterior Cingulate	Sexual	0.045, 0.817
	Bonding	-0.134, 0.487
	Negative	0.291, 0.125
	Neutral	0.058, 0.765
Posterior Cingulate	Sexual	0.023, 0.906
	Bonding	-0.060, 0.759
	Negative	0.484, 0.007**
	Neutral	0.086, 0.658
Medial Frontal Gyrus	Sexual	0.087, 0.653
	Bonding	0.054, 0.783
	Negative	0.486, 0.007**
	Neutral	0.207, 0.279
Nucleus Accumbens	Sexual	-0.026, 0.892
	Bonding	-0.096, 0.622
	Negative	0.475, 0.009**
	Neutral	0.325, 0.084
Caudate	Sexual	0.243, 0.203
	Bonding	0.109, 0.575
	Negative	0.508, 0.005**
	Neutral	0.266, 0.162

Reference:

1. Comninos AN, Wall MB, Demetriou L, Shah AJ, Clarke SA, Narayanaswamy S, Nesbitt A, Izzi-Engbeaya C, Prague JK, Abbara A, et al. Kisspeptin modulates sexual and emotional brain processing in humans. *The Journal of Clinical Investigation.* 2017;127(2):709-19.
